# Discovery and Genomic Characterization of a Novel Ovine Partetravirus and a New Genotype of Bovine Partetravirus

**DOI:** 10.1371/journal.pone.0025619

**Published:** 2011-09-27

**Authors:** Herman Tse, Hoi-Wah Tsoi, Jade L. L. Teng, Xin-Chun Chen, Haiying Liu, Boping Zhou, Bo-Jian Zheng, Patrick C. Y. Woo, Susanna K. P. Lau, Kwok-Yung Yuen

**Affiliations:** 1 Department of Microbiology, The University of Hong Kong, Hong Kong, China; 2 Research Centre of Infection and Immunity, The University of Hong Kong, Hong Kong, China; 3 State Key Laboratory of Emerging Infectious Diseases, The University of Hong Kong, Hong Kong, China; 4 Carol Yu Centre for Infection, The University of Hong Kong, Hong Kong, China; 5 Shenzhen-Hong Kong Institute of Infectious Diseases, Shenzhen Institute of Hepatology, Shenzhen Third People's Hospital, Shenzhen, China; 6 State Key Laboratory for Molecular Virology and Genetic Engineering, Institute of Pathogen Biology, Chinese Academy of Medical Sciences and Peking Union Medical College, Beijing, China; University of Kansas Medical Center, United States of America

## Abstract

Partetravirus is a recently described group of animal parvoviruses which include the human partetravirus, bovine partetravirus and porcine partetravirus (previously known as human parvovirus 4, bovine hokovirus and porcine hokovirus respectively). In this report, we describe the discovery and genomic characterization of partetraviruses in bovine and ovine samples from China. These partetraviruses were detected by PCR in 1.8% of bovine liver samples, 66.7% of ovine liver samples and 71.4% of ovine spleen samples. One of the bovine partetraviruses detected in the present samples is phylogenetically distinct from previously reported bovine partetraviruses and likely represents a novel genotype. The ovine partetravirus is a novel partetravirus and phylogenetically most related to the bovine partetraviruses. The genome organization is conserved amongst these viruses, including the presence of a putative transmembrane protein encoded by an overlapping reading frame in ORF2. Results from the present study provide further support to the classification of partetraviruses as a separate genus in *Parvovirinae*.

## Introduction

The parvoviruses are a group of small, non-enveloped animal viruses with a single-stranded DNA genome between 4 and 6 kb in size [1]. At least 2 open reading frames (ORF) are present in the parvovirus genome, with ORF1 encoding the non-structural proteins and ORF2 encoding the viral capsid proteins. Some parvovirus genomes may contain an additional ORF encoding for other proteins, such as the non-structural protein NP1 found in human bocavirus and bovine parvovirus. Under the current International Committee on Taxonomy of Viruses (ICTV) classification system, the *Parvoviridae* are divided into two subfamilies based on their host range: the *Parvovirinae* which infect vertebrates, and the *Densovirinae* which mainly infect insects and other arthropods. The *Parvovirinae* are further subdivided into 5 genera: the amdoviruses, bocaviruses, dependoviruses, erythroviruses, and the parvoviruses. Novel parvoviruses discovered in recent years, such as the human partetravirus (previously known as human parvovirus 4 or PARV4) [2], are not included in the current classification system, and will be addressed by the ICTV in an upcoming update.

Some human parvoviruses are well-known pathogens associated with a range of diseases in infected patients. Parvovirus B19 (also known as erythrovirus B19) can cause syndromes ranging from erythema infectiosum in children to late intrauterine death in pregnant women. The human bocavirus, a parvovirus discovered only in 2005, was associated with respiratory disease and could be detected in stool of children [3], [4], [5], [6], [7], [8], although clear assessment of its pathogenicity is confounded by the frequent co-detection of other respiratory viruses [9], [10] and its presence in the stool of healthy children. On the other hand, the human partetravirus was initially discovered in patients suffering from acute viral syndrome [2], but its association with clinical disease has yet to be confirmed despite positive PCR detection in blood products, HIV-infected patients, intravenous drug users, transplant patients and blood donors in different localities [11], [12], [13], [14], [15], [16], [17], [18].

Since the discovery of the human partetravirus, related animal viruses have been found in various mammalian species. We first reported the discovery of porcine and bovine partetraviruses (also known as hokoviruses previously), which are novel related animal parvoviruses found in Hong Kong [19]. Closely related porcine partetraviruses have since been found in German wild boar populations [20], while human partetravirus-like viral DNA was detected in the plasma samples of a chimpanzee and baboon in Cameroon [21]. As the genetic distances between human partetravirus and other known parvoviruses at the time of its discovery were relatively large, it was unclear if the human partetravirus had diverged from a human parvovirus ancestor early in the history of parvovirus evolution or if it had diverged from an undiscovered animal parvovirus. Parvovirus evolution is characterized by the development of host-specificity and congruent viral and host phylogenies [22], which has led to the hypothesis of long-term co-evolution of parvoviruses and their hosts. In addition, evolutionary rates can vary significantly among different parvovirus species [23], [24], which complicates evolutionary analysis between distant parvoviruses. Hence, the identification of related partetraviruses in animals has contributed to our knowledge of the evolutionary history of the human partetravirus.

As part of our ongoing program in the discovery of viruses associated with emerging infections, we continued our surveillance for novel partetraviruses in animals closely related to humans. In the present study, bovine and ovine samples were selected for targeted screening, as both domestic cattle and sheep are important food animals in Southern China. PCR screening of the animal samples identified a novel ovine partetraviruses as well as a new genotype of bovine partetravirus, and their nearly full-length genome sequences were obtained. Genomic and phylogenetic analyses of the new viruses confirmed them to be closely related to the previously identified partetraviruses.

## Materials and Methods

### Collection of animal samples

All specimens were collected over a one year period (Sept 2008 to Aug 2009). A total of 110 bovine liver samples, 14 ovine spleen samples and 9 ovine liver samples were collected from local food markets with the assistance of the Veterinary Public Health Section, the Food and Environmental Hygiene Department, and the Agriculture, Fisheries and Conservation Department, the Government of Hong Kong Special Administrative Region (HKSAR). It should be noted that ovine liver and spleen samples were not uniformly sampled throughout the study period due to supply issues. All animals from which the specimens had been obtained have passed the relevant health inspection to certify its meat and other products as fit for human consumption. Precautions taken to avoid cross contamination include the use of disposable scalpels during tissue dissection and the collection of samples only from the centre of each tissue block after surface decontamination. Disposable protective gloves were also used and changed after processing each tissue sample.

### PCR detection of parvoviruses

DNA was extracted from all samples using QIAamp DNA Mini kit (Qiagen) according to manufacturer's instructions, and then subjected to PCR using 2 different sets of screening PCR primers. The primer sequences are: forward primer A 5′- CCCGCKASTACWGGNAARAC -3′ and reverse primer A 5′- CCGTAAYTCKRCCYTCKTCCCA -3′ (targeting a 148 bp fragment); forward primer B 5′-TCTGCTATTGTAATHAARGAYGT-3′ and reverse primer B 5′-AAACACTCTGCRTCRTGRTGYTC-3′ (targeting a 293 bp fragment). The primers were designed from multiple alignments of the nucleotide sequences of VP2 regions of PARV4 and related parvoviruses using our previously described strategy [19]. The PCR mixture (25 µl) contained DNA extracted from samples, PCR buffer (10 mM Tris/HCl pH 8.3, 50 mM KCl, 2 mM MgCl_2_ and 0.01% gelatin), 200 µM of each dNTP and 1.0 U AmpliTaq Gold polymerase (Applied Biosystems). PCR cycling conditions were as follows: hot start at 94°C for 7 min, followed by 50 cycles of 94°C for 1 min, 50°C for 1 min and 72°C for 1 min with a final extension at 72°C for 10 min in an automated thermal cycler (Applied Biosystems). Standard precautions were taken to avoid PCR contamination and no false positive results was observed in negative controls.

PCR products were gel-purified using the QIAquick gel extraction kit (Qiagen). Both strands of the PCR products were sequenced twice with an ABI Prism 3700 DNA Analyser (Life Technologies) by using the PCR primers as sequencing primers. The sequences of the PCR products were searched against known VP2 sequences of parvoviruses in the National Center for Biotechnology Information (NCBI) GenBank database using BLASTN and TBLASTN.

### Genome sequencing

Nearly full-length genome sequences spanning the entire protein-coding regions were determined for 2 bovine and 4 ovine strains of the PARV4-related parvoviruses identified in the present study by using our genome sequencing strategy [19]. Briefly, DNA extracted from the corresponding specimens was used as template and amplified by degenerate primers designed from multiple alignment of PARV4 and related sequences available in NCBI GenBank. Additional primers were designed from the first and subsequent rounds of sequencing. Non-overlapping regions were confirmed by independent PCR and sequencing reactions by using specific primers, and no sequence discrepancies were found between repeated sequencing of any region. Sequences of genome amplification and sequencing primers are available from the authors upon request. For sequencing of the terminal regions, a modified protocol for rapid amplification of cDNA ends was adopted [25]. Sequences were assembled and manually edited to produce final sequences of the viral genomes.

### Phylogenetic and sequence analysis

Open reading frames were located using the ORF Finder tool at NCBI (http://www.ncbi.nlm.nih.gov/projects/gorf/) and by comparison with the genome annotations of PARV4 and partetraviruses. Prediction of transmembrane domains was performed using TMHMM version 2.0 server (http://www.cbs.dtu.dk/services/TMHMM/) [26]. Functional annotation of predicted proteins was performed by BLAST similarity search against annotations in the NCBI RefSeq database, as well as by using the InterProScan search tool (http://www.ebi.ac.uk/Tools/pfa/iprscan/) [27]. Multiple alignments of sequences for phylogenetic analysis were constructed using MUSCLE version 3.8.31 [28], and phylogenetic informative regions were extracted using BMGE [29]. Maximum-likelihood phylogenetic trees were constructed using PHYML version 3 [30], under the best-fit protein evolution model as selected by ProtTest 3 [31]. Recombination detection was performed using bootscan analysis (as implemented in SimPlot) [32] and GARD [33]. Sites under positive selection were inferred by consensus between the single-likelihood ancestor counting (SLAC), fixed effects likelihood (FEL) and random effects likelihood (REL) methods as implemented on the DataMonkey server (http://www.datamonkey.org) [34]. A p-value of <0.1 is considered to be statistically significant for the positive selection analysis.

### Nucleotide sequence GenBank accession numbers

The nucleotide sequences of the nearly full-length genomes of the PARV4-related viruses have been submitted to NCBI GenBank, and are available under the accession numbers JF504697 – JF504702.

## Results

### Detection of parvovirus DNA in bovine and ovine samples

PCR detection of PARV4-like DNA using screening primer set A was positive for 2 out of 110 (1.8%) bovine liver samples, while PCR using screening primer set B was positive for 10 out of 14 (71.4%) ovine spleen samples and 6 out of 9 (66.7%) ovine liver samples. The rate of positive PCR detection is significantly higher in ovine liver samples than in bovine live samples (by Fisher's exact test; *p*<0.001), while no statistically significant difference was noted between ovine liver and ovine spleen samples (by Fisher's exact test; *p*>0.05). Sequencing of the PCR products was performed: sequences from the two positive bovine liver samples showed the highest nucleotide identities (100% and 89%) to the ORF2 of bovine partetravirus HK2 (GenBank accession no. EU200670), and sequences from the positive ovine samples showed the highest nucleotide identity (88%) to the ORF2 of human partetravirus PARV4 strain BONN-14 (GenBank accession no. EU175857). This suggested that two related but distinct strains of partetravirus are present in the two different bovine samples, while a novel partetravirus is present in the ovine samples.

### Genome organization and coding potential

The nearly full-length genomes of 2 strains of bovine partetravirus (5096–5240 nucleotides) and 4 strains of ovine partetravirus (2 from liver samples and 2 from spleen samples) (5249 nucleotides) were determined. Similar to our earlier attempt at sequencing the complete genomes of other PARV4-like animal parvoviruses [19], the obtained genome lengths were shorter than expected, as sequencing of the ends of the genomes was hampered by the presence of extensive hairpin structures. Pairwise comparison of the genome sequences of the parvoviruses was performed. Among the presently identified bovine partetraviruses, HK4 was highly similar (99.3−99.4% nucleotide sequence identity) to the bovine partetraviruses reported previously, while HK5 showed much greater sequence divergence (90.0–90.1% nucleotide sequence identity). The genome sequences of the ovine parvoviruses were 100% identical, and they exhibited 68.2–68.3% nucleotide sequence identities to that of the bovine partetraviruses and around 64.0% nucleotide sequence identities to that of the porcine partetraviruses. Results of further sequence comparison with related parvoviruses were shown in Tables 1 and 2.

**Table 1:** Pairwise ORF1 sequence comparison of bovine and ovine partetraviruses (PtV) with other parvoviruses (PV).

	Partetravirus						Parvovirus	Erythrovirus	Bocavirus (BoV)		Amdovirus	Dependovirus
	Bovine PtV HK1	Bovine PtV HK5	Ovine PtV	Human PtV PARV4 C51-4	Bovine PV 2	Bovine PV 3	Minute virus of mice	Human PV B19	Bovine BoV	Human BoV	Aleutian mink disease virus	Bovine AAV
Porcine PtV HK1	62.9/**67.7**	63.8/**68.1**	63.1/**67.5**	59.6/**55.7**	51.1/**25.4**	45.9/**20.5**	49.1/**20.5**	49.3/**26.3**	47.4/**21.2**	43.2/**20.3**	46.2/**18.0**	47.0/**29.1**
Bovine PtV HK1		89.9/**96.6**	68.0/**75.8**	60.4/**57.9**	49.5/**24.7**	45.4/**19.9**	49.5/**19.9**	48.3/**25.5**	48.8/**20.2**	44.9/**20.0**	47.1/**17.9**	46.7/**28.3**
Bovine PtV HK5			67.5/**77.3**	61.5/**58.0**	50.3/**24.9**	45.3/**19.9**	49.9/**19.9**	48.1/**24.8**	49.5/**20.7**	45.8/**20.8**	46.4/**17.3**	47.6/**28.6**
Ovine PtV				60.1/**58.3**	49.6/**24.9**	45.0/**19.3**	48.9/**19.3**	48.4/**25.8**	47.9/**19.2**	44.6/**19.2**	45.8/**19.0**	46.0/**28.5**
Human PtV PARV4 C51-4					49.6/**24.4**	46.7/**19.5**	48.7/**19.5**	50.7/**25.7**	51.1/**20.7**	45.1/**20.4**	47.5/**17.9**	48.7/**27.5**

Non-bold percentages indicate nucleotide identities and bold percentages indicate amino acid identities. All sequence identities are based on the full-length sequences and calculated using MatGat [54]. GenBank accession numbers are as follows: porcine PtV HK1 – EU200671 [19]; bovine PtV HK1 – EU200669 [19]; bovine PtV HK5 –; ovine PtV–; human PtV PARV4 C51-4 – DQ873387 [55]; bovine PV 2 – AF406966 [25]; bovine PV 3 – AF406967 [25]; minute virus of mice – DQ196317 [56]; human PV B19 – AY044266 [57]; bovine bocavirus – DQ335247 [58]; human bocavirus – FJ858259 [59]; Aleutian mink disease virus – GU108231; bovine adeno–associated virus (AAV) – AY388617 [60].

**Table 2:** Pairwise ORF2 sequence comparison of bovine and ovine partetraviruses (PtV) with related parvoviruses (PV).

	Partetravirus						Parvovirus	Erythrovirus	Bocavirus (BoV)		Amdovirus	Dependovirus
	Bovine PtV HK1	Bovine PtV HK5	Ovine PtV	Human PtV PARV4 C51-4	Bovine PV 2	Bovine PV 3	Minute virus of mice	Human PV B19	Bovine BoV	Human BoV	Aleutian mink disease virus	Bovine AAV
Porcine PtV HK1	65.6/**67.0**	65.4/**66.9**	63.9/**66.6**	64.5/**65.5**	46.2/**23.4**	47.8/**26.7**	45.5/**18.2**	46.2/**26.8**	43.0/**17.9**	43.1/**19.2**	41.5/**15.4**	44.9/**20.7**
Bovine PtV HK1		90.6/**97.7**	68.3/**72.9**	65.8/**66.0**	46.7/**23.1**	48.3/**25.1**	45.7/**14.9**	46.5/**25.7**	43.6/**18.4**	43.7/**19.5**	42.3/**16.2**	45.0/**21.9**
Bovine PtV HK5			69.3/**73.3**	65.7/**67.1**	46.7/**23.1**	48.3/**24.7**	45.3/**14.7**	46.1/**26.3**	43.5/**19.1**	43.8/**19.7**	42.3/**15.8**	45.6/**21.9**
Ovine PtV				65.1/**68.1**	46.2/**22.9**	47.8/**25.1**	45.4/**16.1**	45.9/**26.5**	43.5/**17.6**	43.1/**17.8**	42.2/**14.6**	44.0/**22.2**
Human PtV PARV4 C51-4					47.3/**23.7**	48.5/**25.0**	46.0/**16.4**	47.3/**26.4**	43.9/**18.1**	43.1/**18.1**	43.6/**14.6**	45.3/**22.0**

Non-bold percentages indicate nucleotide identities and bold percentages indicate amino acid identities. All sequence identities are based on the full-length sequences and calculated using MatGat [54]. GenBank accession numbers are as follows: porcine PtV HK1 – EU200671 [19]; bovine PtV HK1 – EU200669 [19]; bovine PtV HK5 –; ovine PtV–; human PtV PARV4 C51-4 – DQ873387 [55]; bovine PV 2 – AF406966 [25]; bovine PV 3 – AF406967 [25]; minute virus of mice – DQ196317 [56]; human PV B19 – AY044266 [57]; bovine bocavirus – DQ335247 [58]; human bocavirus – FJ858259 [59]; Aleutian mink disease virus – NC_001662 [61]; bovine adeno–associated virus (AAV) – AY388617 [60].

All sequenced virus strains possessed a genome organization typical of parvoviruses. There were two large non-overlapping ORFs, with ORF1 encoding a non-structural polyprotein NS1 and ORF2 encoding overlapping VP1/VP2 capsid proteins and a small conserved putative protein. A small non-coding gap of around 115 nucleotides was found between the two ORFs. Inverted terminal repeats were found at the 5′ and 3′ ends of the viral genome. Further sequence analysis was not performed for the bovine partetravirus HK4 due to its high genetic similarity with previously characterized bovine partetraviruses. For bovine partetravirus HK5, the predicted NS1 protein consists of 652 aa and is 73.9 kDa in molecular weight. Conserved sequence features including helicase and ATPase domains are present, in agreement with the non-structural functional role of NS1 in parvovirus replication. The VP1 protein is predicted to contain 931 aa and is 102.8 kDa in molecular weight, which is comparable to that of previously identified bovine partetraviruses and larger than the average molecular weight of VP1 in other parvoviruses. Conserved functional motifs like phospholipase A_2_ were identified in the VP1 unique (VP1u) region. For the ovine parvovirus, the predicted NS1 protein consists of 656 aa and is 74.2 kDa in molecular weight, while its VP1 protein contains 933 aa and is 103.2 kDa in molecular weight. Both proteins contain the same conserved functional motifs as their homologs in bovine partetravirus HK5.

Similar to other partetraviruses, there exists a putative third ORF in a different reading frame within the VP1u region for both bovine partetravirus HK5 and ovine parvovirus. In agreement with previous studies, the phospholipase A_2_ motifs of ORF2 are found in the VP1u region, which is also the most conserved region among partetraviruses [19], [35]. The putative third ORF encodes a small protein containing a single transmembrane helix spanning 20 aa in the centre, with predicted molecular weights of 9.5 kDa in bovine partetravirus HK5 and 9.7 kDa in ovine parvovirus. The aa sequences of this putative protein are conserved among human, bovine, ovine and porcine partetraviruses, with pairwise sequence identities ranging from 59.3% (between porcine partetravirus HK7 and ovine partetravirus) to 96.4% (between bovine partetravirus HK1 and bovine partetravirus HK5).

### Phylogenetic and sequence analysis

**Figure 1:**
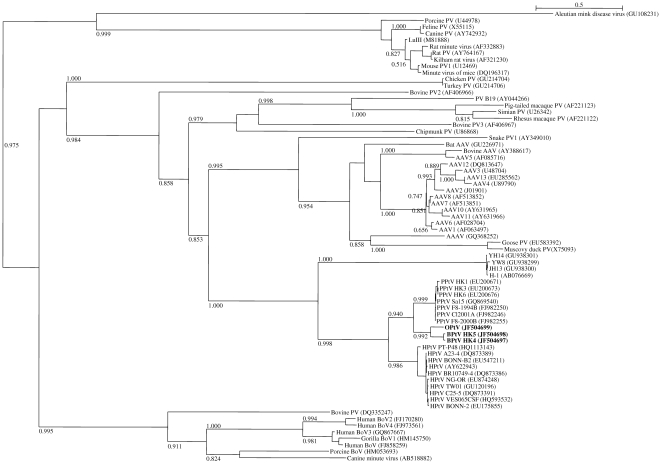
Phylogenetic tree constructed from the full-length aa sequences of NS1 of partetraviruses and other parvoviruses. Approximate likelihood SH-like support values >0.50 are shown besides major branches. NS1 aa sequences of bovine partetravirus HK1 to HK3 are identical to that of bovine partetravirus HK4 and not shown. Scale bar indicates 0.5 inferred substitutions per site. Abbreviations: PPtV, porcine partetravirus; BPtV, bovine partetravirus; OPtV, ovine partetravirus; PV, parvovirus; AAV, adeno-associated virus; BoV, bocavirus.

**Figure 2:**
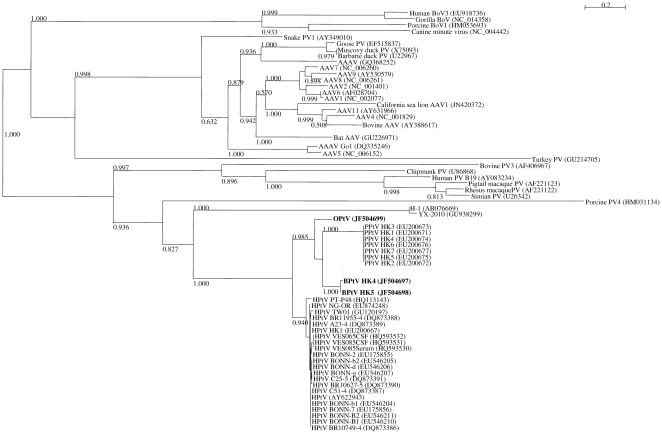
Phylogenetic tree constructed from the full-length aa sequences of VP1 of partetraviruses and other parvoviruses. Approximate likelihood SH-like support values >0.50 are shown besides major branches. VP1 aa sequences of bovine partetravirus HK1 to HK3 are identical to that of bovine partetravirus HK4 and not shown. Scale bar indicates 0.2 inferred substitutions per site. Abbreviations: PPtV, porcine partetravirus; BPtV, bovine partetravirus; OPtV, ovine partetravirus; PV, parvovirus; AAV, adeno-associated virus; BoV, bocavirus.

Phylogenetic inference was conducted on the multiple sequence alignment of full-length NS1 and VP1/2 sequences of the partetraviruses. The phylogenetic trees (Fig. 1 and 2) showed a common topology, and placed the presently identified viruses in the same clade as known partetraviruses. Bovine partetraviruses HK4 and HK5 formed a cluster with known bovine partetraviruses, although the genetic distance between HK5 and the other bovine partetraviruses suggested that it should be considered as a different genotype of bovine partetravirus. The 4 identical strains of ovine parvovirus are clearly shown to be distinct from the other known partetraviruses, and are most closely related to bovine partetraviruses. Based on these results, we recognize these ovine parvoviruses as a new member of the partetraviruses and propose to describe them as ovine partetraviruses. The phylogeny is consistent with the hypothesis of virus-host co-evolution, as the sheep and cattle are both ruminants and are evolutionarily closer to each other than to the other hosts like pigs, chimpanzee and humans.

Bootscan analysis on the genome sequences of PARV4-related viruses highlighted some genomic regions that may have undergone past recombination events between ovine parvovirus and PARV4 (Fig. S1). GARD analysis on the DataMonkey server (http://www.datamonkey.org/) also revealed the presence of 11 potential recombination breakpoints (data not shown), suggesting a multitude of possible past recombination events. However, incongruence testing with the Kishino-Hasegawa (KH) test failed to confirm the presence of phylogenetic incongruence in the partitions proposed by the bootscan and GARD analyses. Hence, it is more likely that the potential recombination signals were false-positives resulting from local differences in evolutionary rates among different genes.

Sequence analysis for sites under positive selection revealed 1 site under positive selection in ORF1 and 13 sites under positive selection in ORF2 (Table 3). No single site was shown to be under positive selection by a consensus of all 3 methods (SLAC, FEL and REL). The overall dN/dS as calculated by the SLAC method were 0.130 for both ORF1 and ORF2. The present results are consistent with the biological functions of the viral proteins, as VP2 (encoded by ORF2) is responsible for receptor binding and would be under greater selection pressure for host adaptation and immune evasion. The significance of these identified sites would require further knowledge of the structure and function of the viral proteins for interpretation.

**Table 3:** Sites under positive selection in partetratvirus ORF1 and ORF2.

Sequence	Codon position	SLAC		FEL		REL	
		dN-dS	p-value	dN-dS	p-value	dN-dS	Bayes factor
ORF1	675	2.993	0.308	0.548	**0.071**	−0.569	1.0
ORF2	115	3.079	0.416	0.441	**0.073**	−0.007	**90.274**
	208	2.399	0.436	0.310	**0.081**	0.129	**359.477**
	209	1.482	0.670	0.193	0.206	0.039	**93.682**
	214	2.730	0.296	0.311	0.145	−0.059	**63.585**
	216	1.820	0.444	0.190	0.140	0.138	**215.139**
	217	3.576	0.212	0.471	**0.085**	−0.065	**61.723**
	226	1.820	0.444	0.206	0.129	0.142	**251.557**
	250	1.820	0.444	0.197	0.239	−0.076	**50.127**
	344	2.758	0.321	0.342	0.117	0.057	**134.893**
	356	1.826	0.453	0.219	0.207	0.036	**93.512**
	358	2.691	0.309	0.335	0.137	−0.075	**58.670**
	382	5.907	**0.099**	1.453	**0.015**	−0.168	38.311
	384	5.251	0.183	1.420	**0.023**	−0.402	14.133

A p-value of <0.1 is considered to be statistically significant for positive selection analysis by SLAC and FEL. A Bayes factor of >50 is considered statistically significant for positive selection by REL.

## Discussion

The present study extends our previous discovery of partetraviruses in domestic animals. Although no clear association has been found between clinical disease and human partetraviruses, the relatively high prevalence of PARV4 in human blood products has raised safety concerns, especially since available evidence appears to support possible parenteral transmission of the virus [14], [36], [37], [38], [39]. Similarly, animal partetraviruses have not been found to be associated with any clinical diseases in the hosts, and are detected frequently in apparently healthy animals [19], [20], [21]. So far, these viruses were mostly detected in liver, spleen, lymph node and bone marrow, suggesting significant tropism for lymphoid tissue, although there is also a report of detecting PARV4 DNA at relatively high frequencies in other organs such as the heart, lungs and kidneys [12]. The development of immune electron microscopy and related techniques can determine the intracellular location and cellular tropism of partetraviruses in infected tissues, and success have been reported with the visualization of PARV4 particles in a high-titre-positive plasma sample [40]. This will aid efforts to target screening for subclinical disease in patients or animals with positive partetravirus detection by PCR or serology.

The understanding of parvovirus evolution has changed greatly over the years. Initially, parvoviruses were thought to evolve slowly relative to RNA viruses due to the utilization of the high-fidelity host DNA polymerase during replication. This was also supported by the very limited sequence diversity of the human pathogenic parvovirus B19 that were known at the time. However, studies examining the evolutionary rate of different parvoviruses suggested that the evolutionary rates of parvoviruses are generally fast and comparable to those of RNA viruses. Moreover, a broad range of rates have been observed for different viruses: 1.7×10^−4^ substitution/site/year (s/s/yr) in canine parvovirus, 9.4×10^−5^ s/s/yr in the closely related feline panleukopenia parvovirus [23], 8.6×10^−4^ s/s/yr in human bocavirus [41], 1×10^−4^ s/s/yr in parvovirus B19 [42]. In particular, it has been inferred that the evolutionary rate were as high as 0.7−7.1×10^−3^ s/s/yr during the time interval from which canine parvovirus emerged. The discovery of additional genotypes of parvovirus B19 has also bolstered the view of parvoviruses as fast-evolving viruses [43], [44].

Nonetheless, questions remain on certain aspects of parvovirus evolution. Although virus-host co-evolution had been proposed as an important mechanism in the emergence of new animal parvoviruses [22], this hypothesis cannot be easily reconciled with the high evolutionary rates observed in diverse parvoviruses, as the evolutionary timescale of the genetic distance between the animal hosts would be several orders of magnitude greater than that of the genetic distance between the corresponding parvoviruses. While the effects of negative selection on parvoviruses highly adapted to their animal host may slow further evolution of the virus, it is unlike to account for the discrepancy between host and virus evolution to a significant degree. In comparison, observations on the emergence of canine parvovirus from feline panleukopenia parvovirus [45] and the experimental cross-species infection of rodent parvoviruses provide more support to the role of cross-species transmission in the evolution of parvoviruses. This would also be more consistent with the degree of recombination seen in diverse parvoviruses [46], which would have required frequent cross-species transmission and co-infection of different parvoviruses in the same host [47].

The present detection of these partetraviruses in organs of otherwise healthy animals suggests the possibility of viral persistence, although comprehensive sampling of other sites such as the respiratory tract would be needed to exclude acute infections. Relatively little is known about persistence of partetraviruses in animal hosts, although the phenomenon is well-described for other parvoviruses. Genomic DNA of human erythrovirus genotype 1 has been found to persist in the synovial membranes of both patients with chronic arthropathy and healthy individuals [48]. A larger study has additionally uncovered information on the distribution and persistence among different genotypes of human erythrovirus, which revealed the epidemiological history of these genotypes in the human population [49]. The exact mechanism for viral persistence has not been fully elucidated for many parvoviruses, though site-specific integration has been described for adeno-associated viruses [50]. Among the partetraviruses, the phenomenon of persistence is best studied in the human partetravirus genotype PARV4, which has been found in the blood, lymphoid tissue and bone marrow of HIV-infected patients [12], [18]. Results from interferon γ enzyme-linked immunospot assays suggested that PARV4 persistence may be present at 26% of hepatitis C virus-positive individuals [51].

From the results of the current and previous studies, it is shown that a diversity of partetraviruses could be found in humans and other animals. As these viruses were only recently discovered, only limited epidemiological and genetic data is available and they are unlikely to reflect their true prevalence and genetic diversity. Nonetheless, at least 3 genotypes have been identified for the human partetravirus [52], [53], which showed some degree of geographic segregation in their circulation. In contrast, the porcine partetraviruses identified in wild boars in Germany were highly similar to the porcine partetraviruses initially discovered in Hong Kong, though it remains possible to distinguish them as two close clusters of viruses on a phylogenetic tree constructed from discontinuous genome sequences [20]. The distinct cluster formation by these related viruses on phylogenetic trees argue for the consideration of the viruses to be classified in a new genus, which has already been suggested in a current ICTV taxonomic proposal. The present discovery of the ovine partetravirus and a new genotype of bovine partetravirus adds to the diversity of known partetraviruses, and should aid future efforts to characterize the evolution and transmission of these related viruses.

## Supporting Information

Figure S1 **Bootscan analysis on the genome sequences of ovine partetravirus and related viruses (porcine partetravirus HK7 (P), human partetravirus HK1 (H), and bovine partetraviruses HK4 and HK5 (B)) using Simplot version 3.5.1.** Consensus threshold of 50% was employed for analysing the bovine partetravirus sequences. Parameters for the analysis are shown in the figure.
(PDF)

## References

[1] Berns K, Parrish CR, Knipe DM, Howley PM (2007). Parvoviridae.. Fields Virology.

[2] Jones MS, Kapoor A, Lukashov VV, Simmonds P, Hecht F (2005). New DNA viruses identified in patients with acute viral infection syndrome.. J Virol.

[3] Allander T, Tammi MT, Eriksson M, Bjerkner A, Tiveljung-Lindell A (2005). Cloning of a human parvovirus by molecular screening of respiratory tract samples.. Proc Natl Acad Sci U S A.

[4] Sloots TP, McErlean P, Speicher DJ, Arden KE, Nissen MD (2006). Evidence of human coronavirus HKU1 and human bocavirus in Australian children.. J Clin Virol.

[5] Catalano-Pons C, Bue M, Laude H, Cattan F, Moulin F (2007). Human bocavirus infection in hospitalized children during winter.. Pediatr Infect Dis J.

[6] Lau SK, Yip CC, Que TL, Lee RA, Au-Yeung RK (2007). Clinical and molecular epidemiology of human bocavirus in respiratory and fecal samples from children in Hong Kong.. J Infect Dis.

[7] Gagliardi TB, Iwamoto MA, Paula FE, Proenca-Modena JL, Saranzo AM (2009). Human bocavirus respiratory infections in children.. Epidemiol Infect.

[8] Moriyama Y, Hamada H, Okada M, Tsuchiya N, Maru H (2010). Distinctive clinical features of human bocavirus in children younger than 2 years.. Eur J Pediatr.

[9] Schildgen O, Muller A, Allander T, Mackay IM, Volz S (2008). Human bocavirus: passenger or pathogen in acute respiratory tract infections?. Clin Microbiol Rev.

[10] Kahn J (2008). Human bocavirus: clinical significance and implications.. Curr Opin Pediatr.

[11] Touinssi M, Reynaud-Gaubert M, Gomez C, Thomas P, Dussol B (2011). Parvovirus 4 in French in-patients: A study of hemodialysis and lung transplant cohorts.. J Med Virol.

[12] Corcioli F, Zakrzewska K, Fanci R, De Giorgi V, Innocenti M (2010). Human parvovirus PARV4 DNA in tissues from adult individuals: a comparison with human parvovirus B19 (B19V).. Virol J.

[13] Simmonds P, Douglas J, Bestetti G, Longhi E, Antinori S (2008). A third genotype of the human parvovirus PARV4 in sub-Saharan Africa.. J Gen Virol.

[14] Schneider B, Fryer JF, Oldenburg J, Brackmann HH, Baylis SA (2008). Frequency of contamination of coagulation factor concentrates with novel human parvovirus PARV4.. Haemophilia.

[15] Fryer JF, Hubbard AR, Baylis SA (2007). Human parvovirus PARV4 in clotting factor VIII concentrates.. Vox Sang.

[16] Longhi E, Bestetti G, Acquaviva V, Foschi A, Piolini R (2007). Human parvovirus 4 in the bone marrow of Italian patients with AIDS.. AIDS.

[17] Fryer JF, Delwart E, Hecht FM, Bernardin F, Jones MS (2007). Frequent detection of the parvoviruses, PARV4 and PARV5, in plasma from blood donors and symptomatic individuals.. Transfusion.

[18] Manning A, Willey SJ, Bell JE, Simmonds P (2007). Comparison of tissue distribution, persistence, and molecular epidemiology of parvovirus B19 and novel human parvoviruses PARV4 and human bocavirus.. J Infect Dis.

[19] Lau SK, Woo PC, Tse H, Fu CT, Au WK (2008). Identification of novel porcine and bovine parvoviruses closely related to human parvovirus 4.. J Gen Virol.

[20] Adlhoch C, Kaiser M, Ellerbrok H, Pauli G (2010). High prevalence of porcine Hokovirus in German wild boar populations.. Virol J.

[21] Sharp CP, LeBreton M, Kantola K, Nana A, Diffo Jle D (2010). Widespread infection with homologues of human parvoviruses B19, PARV4, and human bocavirus of chimpanzees and gorillas in the wild.. J Virol.

[22] Lukashov VV, Goudsmit J (2001). Evolutionary relationships among parvoviruses: virus-host coevolution among autonomous primate parvoviruses and links between adeno-associated and avian parvoviruses.. J Virol.

[23] Shackelton LA, Parrish CR, Truyen U, Holmes EC (2005). High rate of viral evolution associated with the emergence of carnivore parvovirus.. Proc Natl Acad Sci U S A.

[24] Zuo J, Rao J, Xu H, Ma L, Li B (2010). Analysis of the *vp2* gene sequence of a new mutated mink enteritis parvovirus strain in PR China.. Virol J.

[25] Allander T, Emerson SU, Engle RE, Purcell RH, Bukh J (2001). A virus discovery method incorporating DNase treatment and its application to the identification of two bovine parvovirus species.. Proc Natl Acad Sci U S A.

[26] Krogh A, Larsson B, von Heijne G, Sonnhammer EL (2001). Predicting transmembrane protein topology with a hidden Markov model: application to complete genomes.. J Mol Biol.

[27] Quevillon E, Silventoinen V, Pillai S, Harte N, Mulder N (2005). InterProScan: protein domains identifier.. Nucleic Acids Res.

[28] Edgar RC (2004). MUSCLE: multiple sequence alignment with high accuracy and high throughput.. Nucleic Acids Res.

[29] Criscuolo A, Gribaldo S (2010). BMGE (Block Mapping and Gathering with Entropy): a new software for selection of phylogenetic informative regions from multiple sequence alignments.. BMC Evol Biol.

[30] Guindon S, Gascuel O (2003). A simple, fast, and accurate algorithm to estimate large phylogenies by maximum likelihood.. Syst Biol.

[31] Darriba D, Taboada GL, Doallo R, Posada D (2011). ProtTest 3: fast selection of best-fit models of protein evolution..

[32] Lole KS, Bollinger RC, Paranjape RS, Gadkari D, Kulkarni SS (1999). Full-length human immunodeficiency virus type 1 genomes from subtype C-infected seroconverters in India, with evidence of intersubtype recombination.. J Virol.

[33] Kosakovsky Pond SL, Posada D, Gravenor MB, Woelk CH, Frost SD (2006). GARD: a genetic algorithm for recombination detection.. Bioinformatics.

[34] Kosakovsky Pond SL, Frost SD (2005). Not so different after all: a comparison of methods for detecting amino acid sites under selection.. Mol Biol Evol.

[35] Fryer JF, Kapoor A, Minor PD, Delwart E, Baylis SA (2006). Novel parvovirus and related variant in human plasma.. Emerg Infect Dis.

[36] Szelei J, Liu K, Li Y, Fernandes S, Tijssen P (2010). Parvovirus 4-like virus in blood products.. Emerg Infect Dis.

[37] Yang SJ, Hung CC, Chang SY, Lee KL, Chen MY (2011). Immunoglobulin G and M antibodies to human parvovirus 4 (PARV4) are frequently detected in patients with HIV-1 infection.. J Clin Virol.

[38] Lurcharchaiwong W, Chieochansin T, Payungporn S, Theamboonlers A, Poovorawan Y (2008). Parvovirus 4 (PARV4) in serum of intravenous drug users and blood donors.. Infection.

[39] Simmonds P, Manning A, Kenneil R, Carnie FW, Bell JE (2007). Parenteral transmission of the novel human parvovirus PARV4.. Emerg Infect Dis.

[40] Tuke PW, Parry RP, Appleton H (2010). Parvovirus PARV4 visualization and detection.. The Journal of general virology.

[41] Zehender G, De Maddalena C, Canuti M, Zappa A, Amendola A (2010). Rapid molecular evolution of human bocavirus revealed by Bayesian coalescent inference.. Infect Genet Evol.

[42] Shackelton LA, Holmes EC (2006). Phylogenetic evidence for the rapid evolution of human B19 erythrovirus.. J Virol.

[43] Nguyen QT, Wong S, Heegaard ED, Brown KE (2002). Identification and characterization of a second novel human erythrovirus variant, A6.. Virology.

[44] Servant A, Laperche S, Lallemand F, Marinho V, De Saint Maur G (2002). Genetic diversity within human erythroviruses: identification of three genotypes.. J Virol.

[45] Parrish CR (1999). Host range relationships and the evolution of canine parvovirus.. Vet Microbiol.

[46] Shackelton LA, Hoelzer K, Parrish CR, Holmes EC (2007). Comparative analysis reveals frequent recombination in the parvoviruses.. The Journal of general virology.

[47] Battilani M, Balboni A, Ustulin M, Giunti M, Scagliarini A (2011). Genetic complexity and multiple infections with more Parvovirus species in naturally infected cats.. Vet Res.

[48] Soderlund M, von Essen R, Haapasaari J, Kiistala U, Kiviluoto O (1997). Persistence of parvovirus B19 DNA in synovial membranes of young patients with and without chronic arthropathy.. Lancet.

[49] Norja P, Hokynar K, Aaltonen LM, Chen R, Ranki A (2006). Bioportfolio: lifelong persistence of variant and prototypic erythrovirus DNA genomes in human tissue.. Proc Natl Acad Sci U S A.

[50] Hamilton H, Gomos J, Berns KI, Falck-Pedersen E (2004). Adeno-associated virus site-specific integration and AAVS1 disruption.. J Virol.

[51] Simmons R, Sharp C, Sims S, Kloverpris H, Goulder P (2011). High frequency, sustained T cell responses to PARV4 suggest viral persistence in vivo.. J Infect Dis.

[52] Panning M, Kobbe R, Vollbach S, Drexler JF, Adjei S (2010). Novel human parvovirus 4 genotype 3 in infants, Ghana.. Emerg Infect Dis.

[53] Simmonds P, Douglas J, Bestetti G, Longhi E, Antinori S (2008). A third genotype of the human parvovirus PARV4 in sub-Saharan Africa.. The Journal of general virology.

[54] Campanella JJ, Bitincka L, Smalley J (2003). MatGAT: an application that generates similarity/identity matrices using protein or DNA sequences.. BMC Bioinformatics.

[55] Fryer JF, Delwart E, Bernardin F, Tuke PW, Lukashov VV (2007). Analysis of two human parvovirus PARV4 genotypes identified in human plasma for fractionation.. The Journal of general virology.

[56] Besselsen DG, Romero MJ, Wagner AM, Henderson KS, Livingston RS (2006). Identification of novel murine parvovirus strains by epidemiological analysis of naturally infected mice.. The Journal of general virology.

[57] Hokynar K, Soderlund-Venermo M, Pesonen M, Ranki A, Kiviluoto O (2002). A new parvovirus genotype persistent in human skin.. Virology.

[58] Qiu J, Cheng F, Johnson FB, Pintel D (2007). The transcription profile of the bocavirus bovine parvovirus is unlike those of previously characterized parvoviruses.. J Virol.

[59] Dijkman R, Koekkoek SM, Molenkamp R, Schildgen O, van der Hoek L (2009). Human bocavirus can be cultured in differentiated human airway epithelial cells.. J Virol.

[60] Schmidt M, Katano H, Bossis I, Chiorini JA (2004). Cloning and characterization of a Bovine Adeno-Associated Virus.. J Virol.

[61] Bloom ME, Alexandersen S, Perryman S, Lechner D, Wolfinbarger JB (1988). Nucleotide sequence and genomic organization of Aleutian mink disease parvovirus (ADV): sequence comparisons between a nonpathogenic and a pathogenic strain of ADV.. J Virol.

