# Supplementary figures and images for: Discovery and Genomic Characterization of a Novel Ovine Partetravirus and a New Genotype of Bovine Partetravirus
Source: PLoS One. 2011 Sep 27;6(9):e25619. doi: 10.1371/journal.pone.0025619 (PMC3181347; doi:10.1371/journal.pone.0025619)

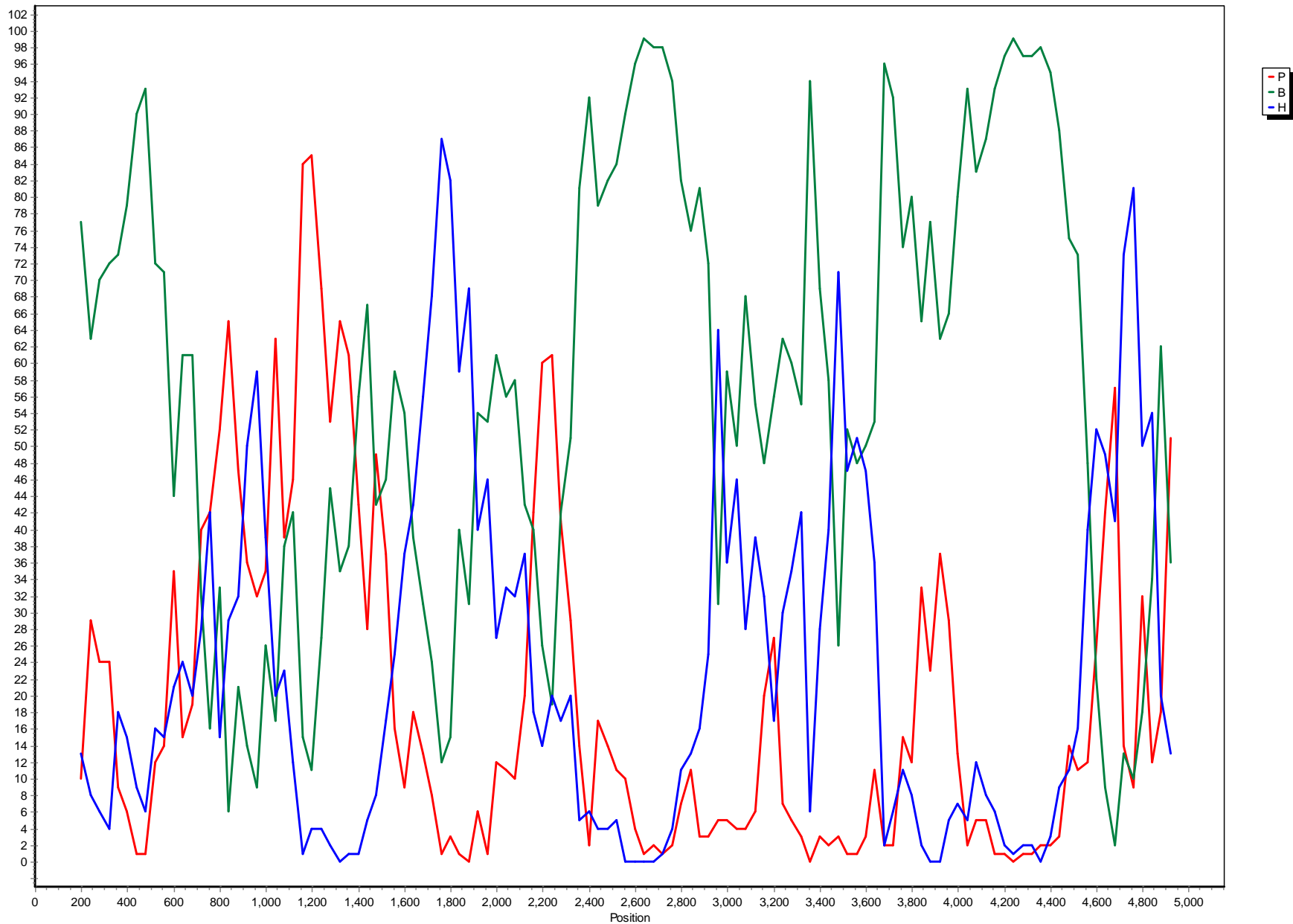

Window: 400 bp, Step: 40 bp, GapStrip: On, Reps: 100, Kimura (2-parameter), T/t: Variable, Neighbor-Joining

Supplement: Figure S1 — Bootscan analysis on the genome sequences of ovine partetravirus and related viruses (porcine partetravirus HK7 (P), human partetravirus HK1 (H), and bovine partetraviruses HK4 and HK5 (B)) using Simplot version 3.5.1. Consensus threshold of 50% was employed for analysing the bovine partetravirus sequences. Parameters for the analysis are shown in the figure. (PDF) [file pone.0025619.s001.pdf]
